# Supplementary material for: Selectively Targeting Breast Cancer Stem Cells by 8-Quinolinol and Niclosamide
Source: Int J Mol Sci. 2022 Oct 4;23(19):11760. doi: 10.3390/ijms231911760 (PMC9570236; doi:10.3390/ijms231911760)
Supplement: Supplementary file 1 [file ijms-23-11760-s001.zip › ijms-1895815-supplementary.pdf]

Selectively targeting breast cancer stem cells by 8-quinolinol and niclosamide

Patricia Cámara-Sánchez[1,2,3], Zamira V. Díaz-Riascos[1,2,3], Natalia García-Aranda[1,2,3], Petra Gener[2,3], Joaquin Seras-Franzoso[2,3], Micaela Giani-Alonso[1,2], Miriam Royo[3,4], Esther Vázquez[3,5], Simó Schwartz Jr.[2,3], Ibane Abasolo[1,2,3]*

1. MATERIALS

Table S1. Summary of anti-CSC drugs tested in vitro. Representation of the chemical structure composition, name (drug abbreviation into brackets), CAS number and bibliographical references of the anti-CSC drugs selected in this work.

Anti-CSC drug		Anti-CSC drug	
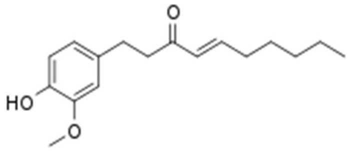<br>**6-shogaol** (6-SHO)<br>555-66-8	[1,2]	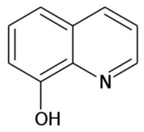<br>**8-quinolinol** (8Q)<br>148-24-3	[3]
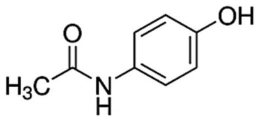<br>**Acetaminophen** (ACE)<br>03-90-2	[4,5]	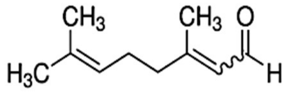<br>**Citral** (CIT)<br>5392-40-5	[6,7]
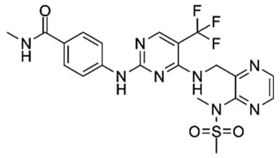<br>**Defactinib** (DFT)<br>1073154-85-4	[8,9]	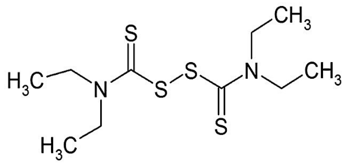<br>**Disulfiram** (DSF)<br>97-77-8	[10–12]

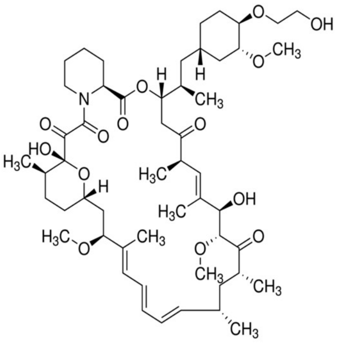<br>**Everolimus** (EVE)<br>159351-69-6	[13,14]	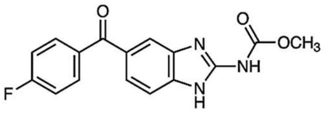<br>**Flubendazole** (FLU)<br>31430-15-6	[15–17]
		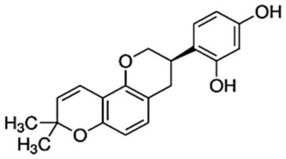<br>**Glabridin** (GLA)<br>59870-68-7	[18,19]
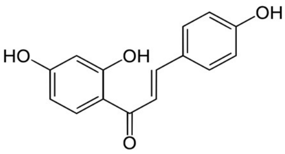<br>**Isoliquiritigenin** (ISO)<br>961-29-5	[20,21]	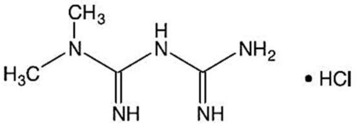<br>**Metformin hydrochloride** (MET)<br>1115-70-4	[22,23]
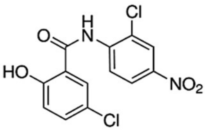<br>**Niclosamide** (NCS)<br>50-65-7	[24,25]	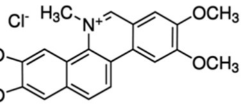<br>**Nitidine chloride** (NTC)<br>13063-04-2	[26,27]
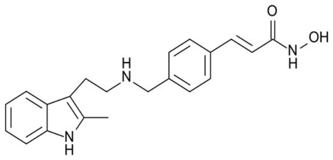<br>**Panobinostat** (PNB)<br>404950-80-7	[28,29]	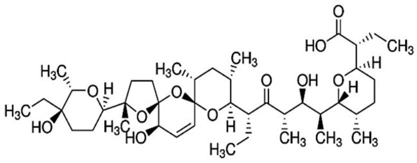<br>**Salinomycin** (SAL)<br>53003-10-4	[29–31]
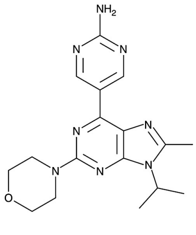<br>**VS-5584** (VS)<br>1246560-33-7	[32,33]	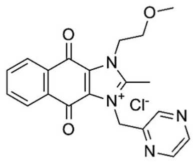<br>**YM-155 hydrochloride** (YM)<br>355406-09-6	[34,35]

2. RESULTS

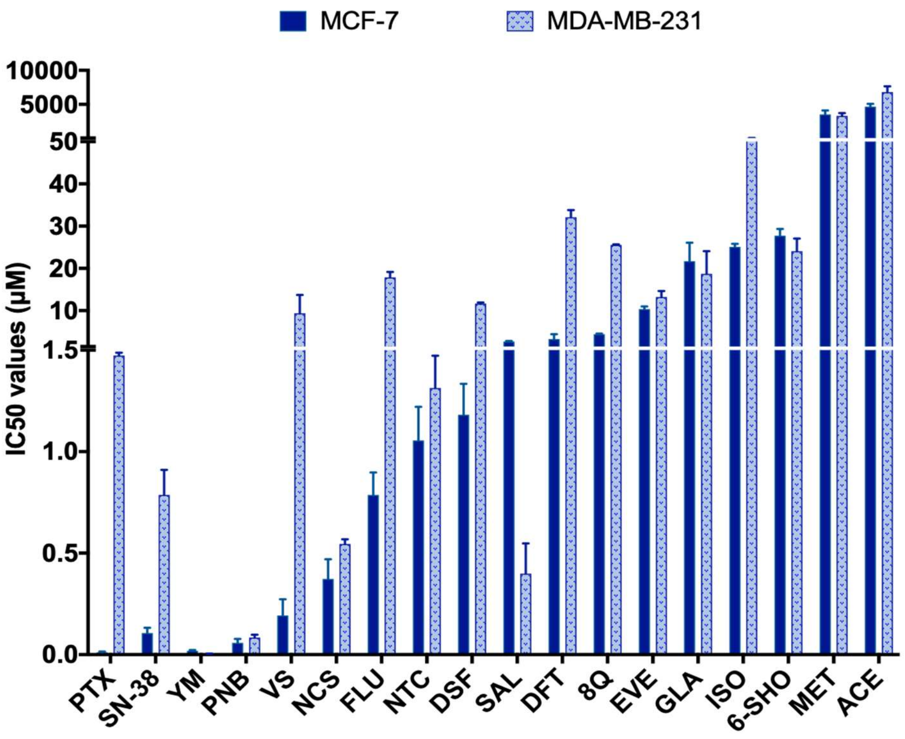

Figure S1. Differential responses of MCF-7 and MDA-MB-231 cell lines to tested compounds. Both cell lines were treated with increasing concentrations of selected drugs for 72 h. Afterwards, IC_{50} values for each compound in both cell lines were calculated. IC_{50} values are represented as the mean ± SEM of three independent experiments.

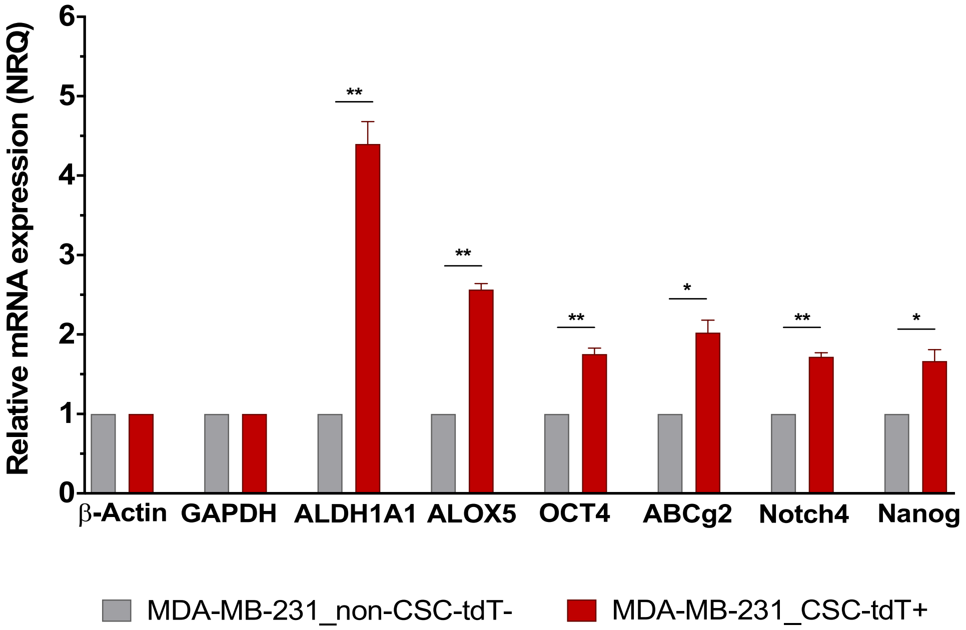

Figure S2. Stem cell-like gene expression profile of enriched CSC and non-CSC subpopulations from MDA-MB-231 fluorescent model measured by qRT-PCR. The stem cell phenotype of tdTomato+ cells was confirmed by a significative overexpression of all the stem cell markers analysed. Results are expressed as NRQ (normalized relative quantities) mean ± SEM (n ≥ 3).

A

Cell number	i.m.f.p. Tumor incidence [Mice (%)]	
	CSC	Non-CSC
100,000	5/5 (100)	5/5 (100)
10,000	5/5 (100)	5/5 (100)
1,000	5/5 (100)	1/5 (20)

B

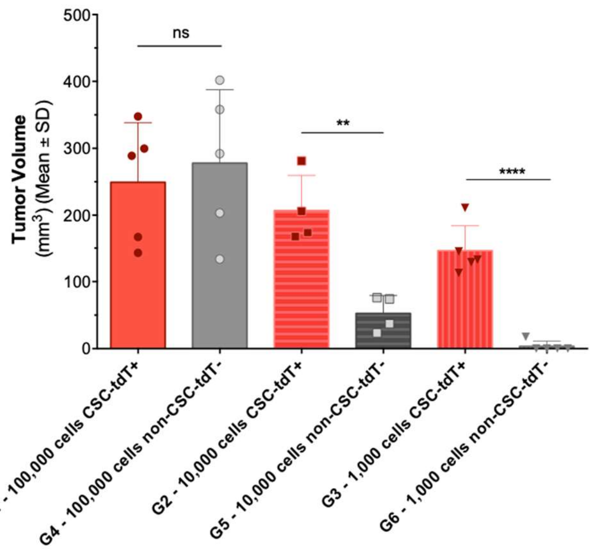

C

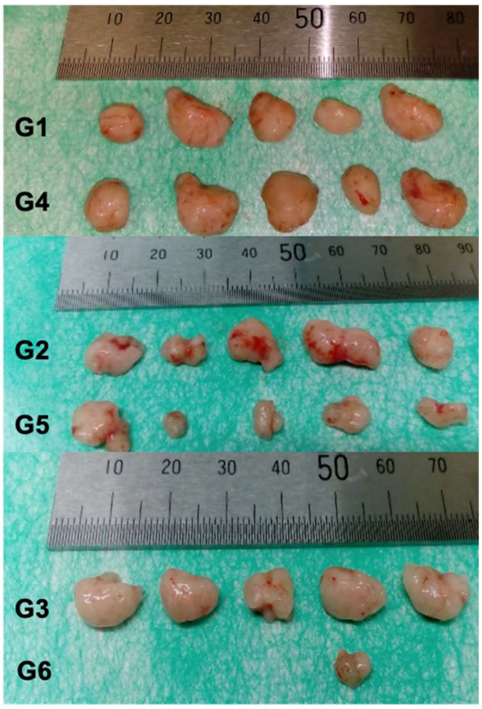

Figure S3. Tumor initiation capacity of tdTomato+ and tdTomato- in HCC-1806.RedFluc.ALDH1A1-tdTomato model. A) Mice were inoculated i.m.f.p. with 100,000, 10,000 and 1,000 of tdTomato+ or of tdTomato− cells and tumor indicidence was evaluated 28 days post-inoculation. B) Ex vivo tumor volumes at 28 days post-inoculation. C) Representative image of the size of excised tumors at 28 days post-inoculation. Differences were regarded as statistically significant (non-parametric Kruskal–Wallis test and unpaired students's t-post test) when p-value was smaller than 0.01 (**).

A

PTX (µM)	*8Q (µM)*	*Combination ratio*	*CI values*
0.02	1.5625	1 : 80	0.13
	3.125	1 : 150	0.11
	6.25	1 : 300	0.15
	12.5	1 : 625	0.23
	25	1 : 1250	0.72
	50	1 : 2500	0.56
	100	1 : 5000	0.53
	200	1 : 10000	0.65
1.3E-04	50	1 : 400000	0.78
6.4E-04		1 : 78000	0.65
3.2E-03		1 : 16000	0.61
0.016		1 : 3000	0.62
0.08		1 : 625	0.88
0.4		1 : 125	2.04
2		1 : 25	4.92
10		1 : 5	1.7E-08

B

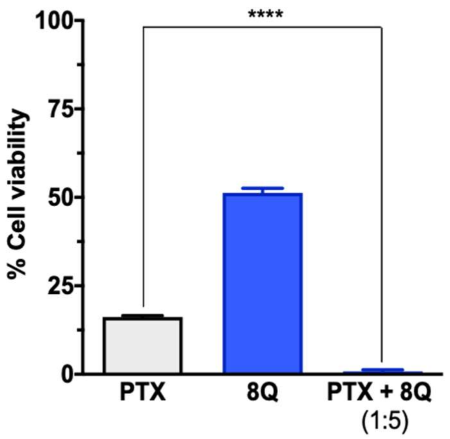

C

PTX (µM)	*NCS (µM)*	*Combination ratio*	*CI values*
0.02	0.625	1 : 30	0.24
	1.25	1 : 60	0.33
	2.5	1 : 125	0.42
	5	1 : 250	0.46
	10	1 : 500	0.36
	20	1 : 1000	0.68
	40	1 : 2000	1.49
	80	1 : 4000	1.13
1.3E-04	4	1 : 30000	0.30
6.4E-04		1 : 6000	0.27
3.2E-03		1 : 1250	0.33
0.016		1 : 250	0.39
0.08		1 : 50	0.94
0.4		1 : 10	4.37
2		1 : 2	2.49
10		1 : 0.4	0.17

D

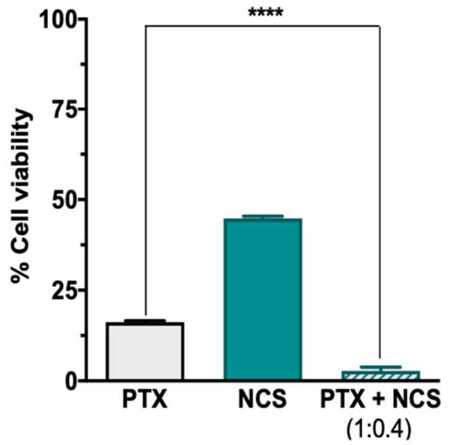

Figure S4. The anti-CSC drugs 8Q and NCS displayed a synergistic inhibition of cell viability when combined with the chemotherapeutic drug PTX in HCC-1806 cell line. Heat maps of **(A)** PTX with 8Q and **(C)** PTX with NCS combined treatments. Graphs show representative results of cell viability (%) when cells were treated with **(B)** PTX and 8Q or **(D)** PTX and NCS, as individual therapy and in combination at 1:5 or 1:0.4 ratios, respectively. Data is represented as the mean ± SEM of three independent experiments.

A

PTX (μM)	*8Q (μM)*	*Combination ratio*	*CI values*
0.005	0.39063	1 : 80	1.69
	0.78125	1 : 150	1.59
	1.5625	1 : 300	1.44
	3.125	1 : 625	1.30
	6.25	1 : 1250	0.02
	12.5	1 : 2500	0.05
	25	1 : 5000	0.05
	50	1 : 10000	0.10
3.1E-03	8	1 : 2500	0.06
6.3E-03		1 : 1000	0.05
0.0125		1 : 600	1.49
0.025		1 : 300	1.15
0.05		1 : 160	1.33
0.1		1 : 80	1.47
0.2		1 : 40	2.24
0.4		1 : 20	2.59

B

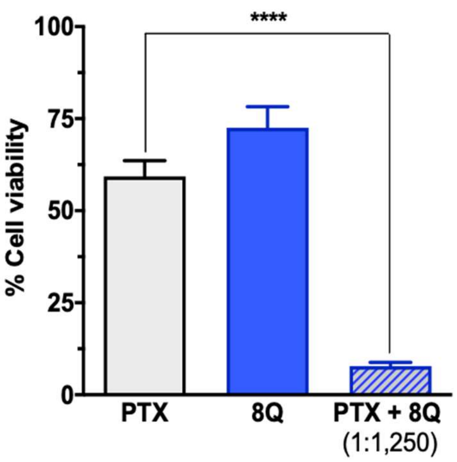

C

PTX (μM)	*NCS (μM)*	*Combination ratio*	*CI values*
0.005	0.15625	1 : 30	1.25
	0.3125	1 : 60	1.10
	0.625	1 : 125	0.69
	1.25	1 : 250	0.51
	2.5	1 : 500	0.17
	5	1 : 1000	0.12
	10	1 : 2000	0.15
	20	1 : 4000	0.16
3.1E-03	2	1 : 600	0.18
6.3E-03		1 : 300	0.21
0.0125		1 : 160	0.30
0.025		1 : 80	0.56
0.05		1 : 40	1.13
0.1		1 : 20	2.08
0.2		1 : 10	3.51
0.4		1 : 5	3.99

D

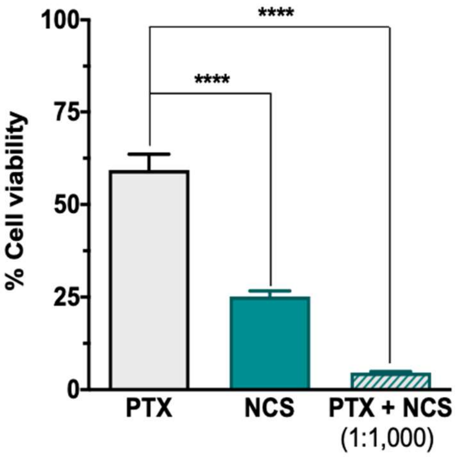

Figure S5. Combination of 8Q or NCS anti-CSC drugs with PTX enhances their synergistic cytotoxic effect in MDA-MB-468 cells. Heat maps of **(A)** PTX with 8Q and **(C)** PTX with NCS combined studies. **(B, D)** Synergism of the combination of anti-CSC drugs with PTX at selected ratios in MDA-MB-468 cells, represented as the % of cell viability obtained when cells are treated with **(B)** PTX and 8Q or **(D)** PTX and NCS, alone and in combination at 1:1,250 or 1:1,000 ratios, respectively. Data is represented as the mean ± SEM of three independent experiments.

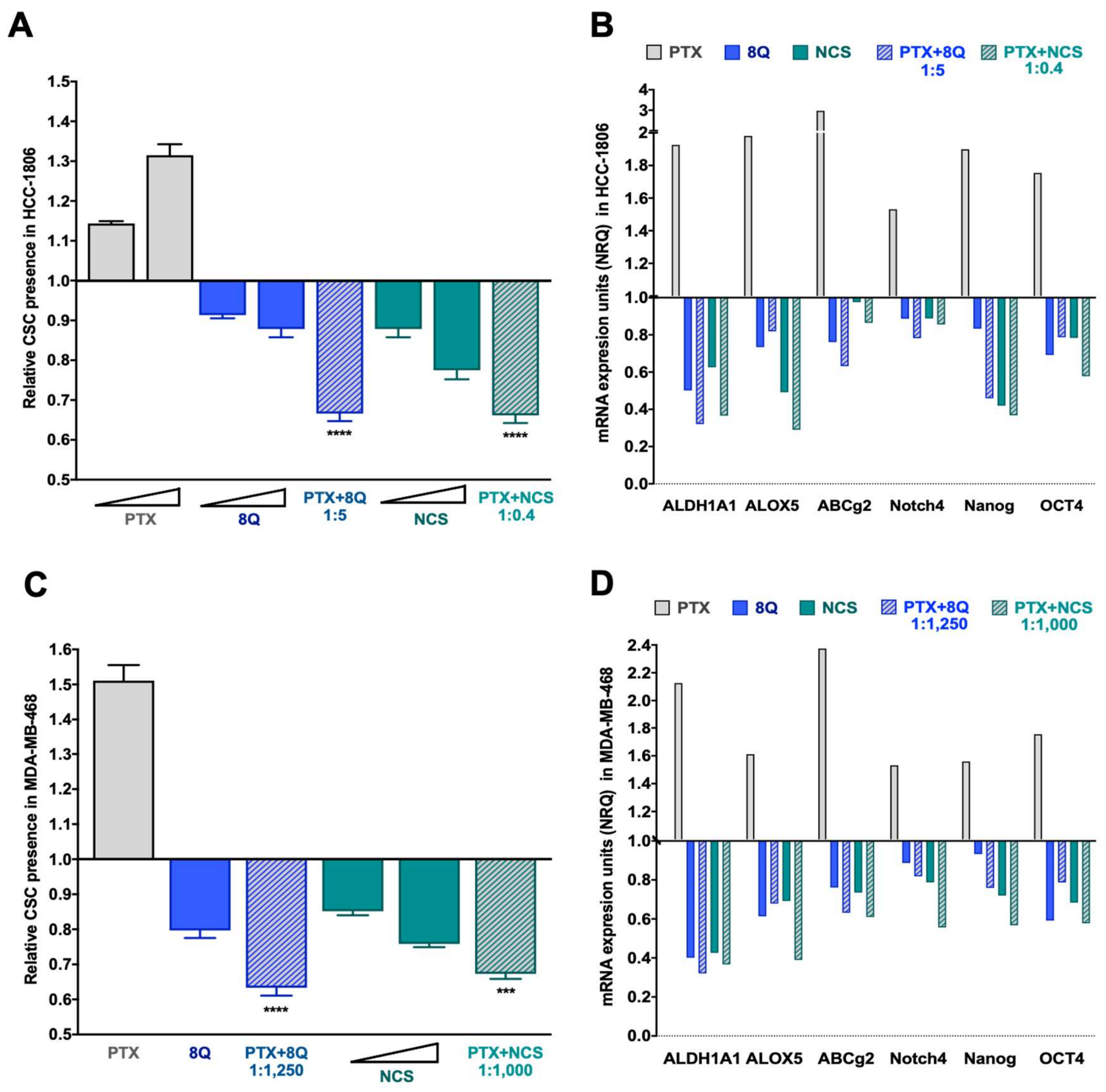

Figure S6. Anti-CSC activity of 8Q and NCS in combination with PTX in TNBC fluorescent CSC models. Relative CSC-tdTomato+ presence in **(A)** HCC-1806 and **(C)** MDA-MB-468 fluorescent models, determined by flow cytometry and referred to control condition. Values below, equal or above 1 indicate reduction, maintenance or increase of CSC-tdTomato+, respectively. Data is represented as the mean ± SEM of three independent experiments. Statistic t-test analysis were performed comparing combination treatments with individual anti-CSC drugs at the corresponding equivalent drug dose. Changes in the stem cell gene expression profile of **(B)** HCC-1806 and **(D)** MDA-MB-468 models determined by quantitative RT-PCR. Results are expressed as normalized relative quantities (NRQ) and referred to control condition. Concentrations tested for drugs in the HCC-1806 cell line were 0.0025 and 5 μM for PTX, 25 and 50 μM for 8Q, 2 and 5 μM for NCS, while for MDA-MB-468 were 0.005 for PTX, 6.25 μM for 8Q, 2.5 and 5 μM for NCS, and the corresponding combined ratios.

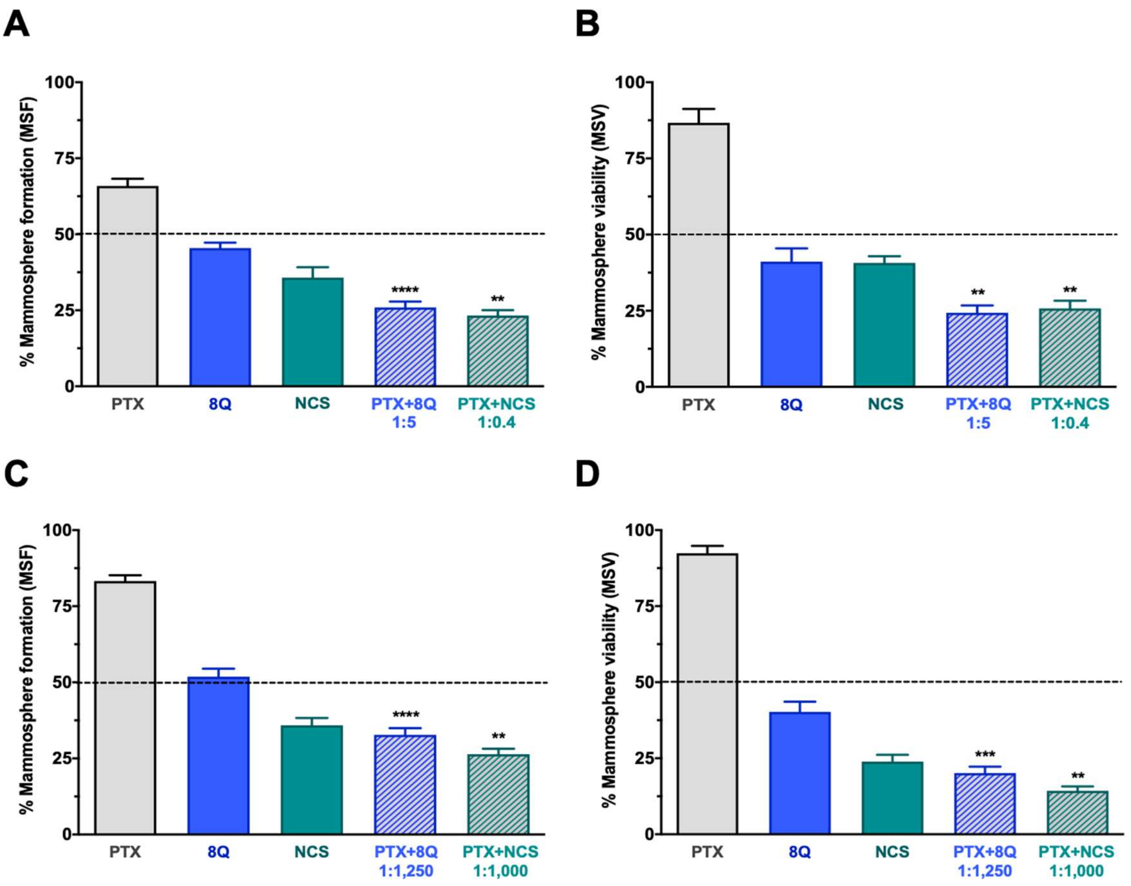

Figure S7. Combination of 8Q or NCS anti-CSC drugs with PTX enhances their synergistic anti-CSC activity in low attachment conditions in other TNBC cell lines. Efficacy of 8Q and NCS, alone and in combination in reducing mammosphere-forming efficiency (MSF) in both **A)** HCC-1806 and **C)** MDA-MB-468 cells, and in affecting mammosphere viability (MSV) in **B)** HCC-1806 and in **D)** MDA-MB-468 cell lines. Data is represented as the mean ± SEM of three independent experiments and referred to non-treated control condition. Drug concentrations used PTX 10 µM, 8Q 50 µM and NCS 4 µM in HCC-1806 cells, while for MDA-MB-468 cells were 0.005 µM, 6.25 µM and 5 µM, respectively. Statistical analysis of combined therapy in comparison with individual anti-CSC treatments are shown in black asterisks.

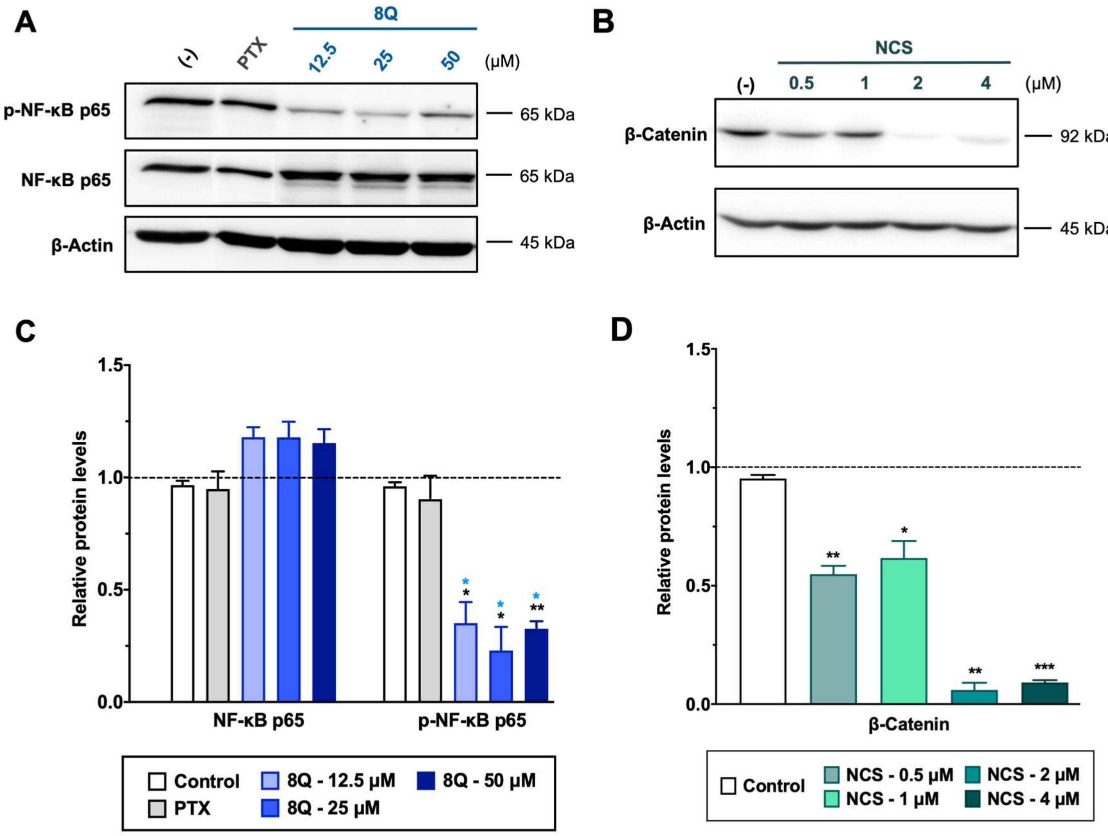

Figure S8. Effect of the combination of 8Q or NCS with PTX in NF-κB and Wnt/β-catenin signaling pathways in MDA-MB-231 cells. A) Representative Western blots of total and phosphorylated NF-κB (p-NF-κB) p65 protein levels upon treatment with different concentrations of 8Q. **B)** Representative Western blots of β-Catenin levels after treatment with increasing concentrations of NCS. The β-actin protein expression level was used as loading control. **C-D)** Graphs represent the quantification band intensity signal referred to β-actin expression, represented as mean ± SEM of three independent experiments. Statistic t-test analysis of anti-CSC drug therapy was performed in comparison with control non-treated cells (black) as well as with PTX treatment (blue).

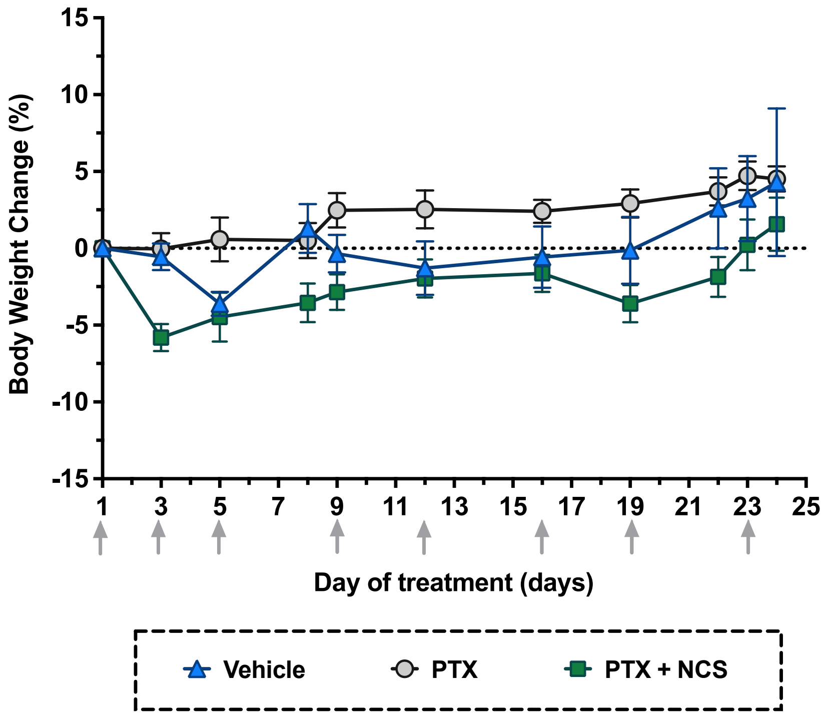

Figure S9. Body weight changes from all three-study groups throughout the experiment. Determination of body weight change referred to the initial weight of mice (before starting the treatments). Results are expressed as mean ± SEM, n≥4.

3. REFERENCES:

1. Ray A.; Vasudevan S.; Sengupta S. 6-Shogaol inhibits breast cancer cells and stem cell-like spheroids by modulation of Notch signaling pathway and induction of autophagic cell death. PLoS One. **2015**, *10* ,1–23.
2. Wu C.H.; Hong B.H.; Ho C.T.; Yen G.C. Targeting cancer stem cells in breast cancer: Potential anticancer properties of 6-shogaol and pterostilbene. *J Agric Food Chem*. **2015**, *63*, 2432–41.
3. Zhou J.; Zhang H.; Gu P.; Margolick J.B.; Yin D.; Zhang Y. Cancer stem/progenitor cell active compound 8-quinolinol in combination with paclitaxel achieves an improved cure of breast cancer in the mouse model. *Breast Cancer Res Treat*. **2009,** *115*,269–77.
4. Ahmed M.; Jinks N.; Babaei-Jadidi R.; Kashfi H.; Castellanosuribe M.; May S.T.; Mukherjee A.; Nateri A.S. Repurposing antibacterial AM404 as a potential anticancer drug for targeting colorectal cancer stem-like cells. *Cancers (Basel)*. **2020**, *12*, 106.
5. Takehara M.; Hoshino T.; Namba T.; Yamakawa N.; Mizushima T. Acetaminophen-induced differentiation of human breast cancer stem cells and inhibition of tumor xenograft growth in mice. *Biochem Pharmacol*. **2011**, *81*, 1124–35.
6. Nigjeh S.E.; Yeap S.K.; Nordin N.; Rahman H.; Rosli R. In vivo anti-tumor effects of citral on 4T1 breast cancer cells via induction of apoptosis and downregulation of aldehyde dehydrogenase activity. *Molecules*. **2019**, *24*, 3241.

7. Thomas M.L.; de Antueno R.; Coyle K.M.; Sultan M.; Cruickshank B.M.; Giacomantonio M.A.; Giacomantonio C.A.; Duncan R.; Marcato P. Citral reduces breast tumor growth by inhibiting the cancer stem cell marker ALDH1A3. *Mol Oncol*. **2016**, *10*, 1485–96.
8. Kolev V.N.; Tam W.F.; Wright Q.G.; McDermott S.P.; Vidal C.M.; Shapiro I.M.; Xu Q.; Wicha M.S.; Pachter J.A.; Weaver D.T. Inhibition of FAK kinase activity preferentially targets cancer stem cells. *Oncotarget*. **2017**, 8, 51733–47.
9. Navas T.; Pfister T.D.; Colantonio S.; Aziz A.; Dieckman L.; Saul R.G., Kaczmarczyk J.; Borgel S.; Alcoser S,Y,: Hollingshead M.G.; et al. Novel antibody reagents for characterization of drug- and tumor microenvironment-induced changes in epithelial-mesenchymal transition and cancer stem cells. *PLoS One*. **2018**, 13, 1–25.
10. Yip N.C.; Fombon I.S.; Liu P.; Brown S.; Kannappan V.; Armesilla A.L.; Xu B.; Cassidy J.; Darling J.L.; Wang W. Disulfiram modulated ROS-MAPK and NFB pathways and targeted breast cancer cells with cancer stem cell-like properties. *Br J Cancer*. **2011**, *104*, 1564–74.
11. Kim Y.J.; Kim J.Y.; Lee N.; Oh E.; Sung D.; Cho T.M.; Seo J.H. Disulfiram suppresses cancer stem-like properties and STAT3 signaling in triple-negative breast cancer cells. *Biochem Biophys Res Commun*. **2017**, *486*, 1069–76.
12. Yang Z.; Guo F.; Albers A.E.; Sehouli J.; Kaufmann A.M. Disulfiram modulates ROS accumulation and overcomes synergistically cisplatin resistance in breast cancer cell lines. *Biomed Pharmacother*. **2019**, *113*, 108727.
13. Yunokawa M.; Koizumi F.; Kitamura Y.; Katanasaka Y.; Okamoto N.; Kodaira M.; Yonemori K.; Shimizu C.; Ando M.; Masutomi K.; et al. Efficacy of everolimus, a novel mTOR inhibitor, against basal-like triple-negative breast cancer cells. *Cancer Sci.* **2012**; *103*, 1665–71.
14. Chen L.; Yang G.; Dong H. Everolimus reverses palbociclib resistance in ER+ human breast cancer cells by inhibiting phosphatidylinositol 3-kinase(PI3K)/Akt/ mammalian target of rapamycin (mTOR) pathway. *Med Sci Monit***.** **2019**, *25*:77–86.
15. Oh E.; Kim Y.J.; An H.; Sung D.; Cho T.M.; Farrand L.; Jang S.; Seo J.H.; Kim J.Y. Flubendazole elicits anti-metastatic effects in triple-negative breast cancer via STAT3 inhibition. *Int J Cancer*. **2018**, *143*:1978–93.
16. Kim Y.J.; Sung D.; Oh E.; Cho Y.; Cho T.M.; Farrand L.; Seo J.H.; Kim J.Y. Flubendazole overcomes trastuzumab resistance by targeting cancer stem-like properties and HER2 signaling in HER2-positive breast cancer. *Cancer Lett*. **2018**, *412*:118–30.
17. Hou Z.J.; Luo X.; Zhang W.; Peng F.; Cui B.; Wu S.J.; Zheng F.M.; Xu J.; Xu L.Z.; Long Z.J.; et al. Flubendazole, FDA-approved anthelmintic, targets breast cancer stem-like cells. *Oncotarget*. **2015**, *6*, 6326–40.
18. Jiang F.; Li Y.; Mu J.; Hu C.; Zhou M.; Wang X.; Si L.; Ning S.; Li Z. Glabridin inhibits cancer stem cell-like properties of human breast cancer cells: An epigenetic regulation of miR-148a/SMAd2 signaling. *Mol Carcinog*. **2016**; *55*, 929–40.
19. Qian J.; Xia M.; Liu W.; Li L.; Yang J.; Mei Y.; Meng Q.; Xie Y. Glabridin resensitizes p-glycoprotein-overexpressing multidrug-resistant cancer cells to conventional chemotherapeutic agents. *Eur J Pharmacol*. **2019**, *852*, 231–43.
20. Lin P.H.; Chiang Y.F.; Shieh T.M.; Chen H.Y.; Shih C.K.; Wang T.H.; Wang K.L.; Huang T.C.; Hong Y.H.; Li S.C.; et al. Dietary compound isoliquiritigenin, an antioxidant from licorice, suppresses triple-negative breast tumor growth via apoptotic death program activation in cell and xenograft animal models. *Antioxidants*. **2020**, *9*, 228.

21. Peng F.; Tang H.; Liu P.; Shen J.; Guan X.; Xie X.; Gao J.; Xiong L.; Jia L.; Chen J.; Peng C. Isoliquiritigenin modulates MIR-374a/PTEN/Akt axis to suppress breast cancer tumorigenesis and metastasis. *Sci Rep*. **2017**, 7, 9022.
22. Jung JW, Park SB, Lee SJ, Seo MS, Trosko JE, Kang KS. Metformin represses self-renewal of the human breast carcinoma stem cells via inhibition of estrogen receptor-mediated OCT4 expression. *PLoS One*. **2011**, *6*, 1–9.
23. Vazquez-Martin A.; Oliveras-Ferraros C.; Del Barco S.; Martin-Castillo B.; Menendez J.A. The anti-diabetic drug metformin suppresses self-renewal and proliferation of trastuzumab-resistant tumor-initiating breast cancer stem cells. *Breast Cancer Res Treat*. **2011**, *126*, 355–64.
24. Liu J.; Chen X.; Ward T.; Pegram M.; Shen K. Combined niclosamide with cisplatin inhibits epithelial-mesenchymal transition and tumor growth in cisplatin-resistant triple-negative breast cancer. *Tumor Biol*. **2016**, *37*, 9825–35.
25. Wang Y.C.; Chao T.K.; Chang C.C.; Yo Y.T.; Yu M.H.; Lai H.C. Drug screening identifies niclosamide as an inhibitor of breast cancer stem-like cells. *PLoS One*. **2013**, 8, e74538.
26. Sun M.; Zhang N.; Wang X.; Li Y.; Qi W.; Zhang H.; Li Z.; Yang Q. Hedgehog pathway is involved in nitidine chloride induced inhibition of epithelial-mesenchymal transition and cancer stem cells-like properties in breast cancer cells. *Cell Biosci*. **2016**, *6*, 1–14.
27. Pan X.; Han H.; Wang L.; Yang L.; Li R.; Li Z.; Liu J, Zhao Q.; Qian M.; Liu M.; et al. Nitidine Chloride inhibits breast cancer cells migration and invasion by suppressing c-Src/FAK associated signaling pathway. *Cancer Lett*. **2011**, *313*, 181–91.
28. Qin G.; Li Y.; Xu X.; Wang X.; Zhang K.; Tang Y.; Qiu H.; Shi D.; Zhang C.; Long Q.; et al. Panobinostat (LBH589) inhibits Wnt/β-catenin signaling pathway via upregulating APCL expression in breast cancer. *Cell Signal*. **2019**, *59*, 62–75.
29. Kai M.; Kanaya N.; Wu S.V.; Mendez C.; Nguyen D.; Luu T.; Chen S. Targeting breast cancer stem cells in triple-negative breast cancer using a combination of LBH589 and salinomycin. *Breast Cancer Res Treat*. **2015**, *151*, 281–94.
30. Tyagi M.; Patro B.S. Salinomycin reduces growth, proliferation and metastasis of cisplatin resistant breast cancer cells via NF-kB deregulation. *Toxicol Vitr*. **2019**, *60*, 125–33.
31. Kamlund S.; Janicke B.; Alm K.; Oredsson S. Salinomycin treatment specifically inhibits cell proliferation of cancer stem cells revealed by longitudinal single cell tracking in combination with fluorescence microscopy. *Appl Sci*. **2020**, *10*, 4732.
32. Kolev V.N.; Wright Q.G.; Vidal C.M.; Ring J.E.; Shapiro I.M.; Ricono J.; Weaver D.T.; Padval M.V.; Pachter J.A. Xu Q. PI3K/mTOR dual inhibitor VS-5584 preferentially targets cancer stem cells. *Cancer Res*. **2015**; *75*, 446–55.
33. Hart S.; Novotny-Diermayr V.; Goh K.C.; Williams M.; Tan Y.C.; Ong L.C.; Cheong A.; Ng B.K.; Amalini C.; Madan B.; et al. VS-5584, a novel and highly selective PI3K/mTOR kinase inhibitor for the treatment of cancer. *Mol Cancer Ther*. **2013**, *12*, 151–61.
34. Ookura M.; Fujii T.; Yagi H.; Ogawa T.; Kishi S.; Hosono N.; Shigemi H.; Yamauchi T.; Ueda T.; Yoshida A. YM155 exerts potent cytotoxic activity against quiescent (G0/G1) multiple myeloma and bortezomib resistant cells via inhibition of survivin and Mcl-1. *Oncotarget*. **2017**, *8*, 111535–50.
35. Véquaud E.; Séveno C.; Loussouarn D.; Engelhart L.; Campone M.; Juin P.; Barillé-Nion S. YM155 potently triggers cell death in breast cancer cells through an autophagy-NF-kB network. *Oncotarget*. **2015**, *6*, 13476–86.
